# Supplementary material for: The magnitude of mental distress and associated factors among a school of medicine and college of health sciences students at Debre Markos University, 2021
Source: PLoS One. 2022 Sep 28;17(9):e0275120. doi: 10.1371/journal.pone.0275120 (PMC9518916; doi:10.1371/journal.pone.0275120)
Supplement: S1 File — (DOCX) [file pone.0275120.s001.docx]

**Sociodemographic characteristics**

| No. | Questionnaire and filters | Coding categories | Code |
| --- | --- | --- | --- |
| 101 | How old are you? | Age in years(_____) |  |
| 102 | Sex | Male | 1 |
|  |  | Female | 2 |
| 103 | What is your religion | Orthodox | 1 |
|  |  | Muslim | 2 |
|  |  | Protestant | 3 |
|  |  | Catholic | 4 |
|  |  | Others specify-------- | 5 |
| 104 | Do you take part in religious practice | Always | 1 |
|  |  | Often | 2 |
|  |  | Some times | 3 |
|  |  | Never | 4 |
| 105 | What is your marital status now? | Single | 1 |
|  |  | Married | 2 |
|  |  | Divorced | 3 |
|  |  | Widowed | 4 |
| 106 | What is your ethnicity | Amhara | 1 |
|  |  | Oromia | 2 |
|  |  | Tigre | 3 |
|  |  | Others specify-------- | 4 |
| 107 | What is your current family place of residence? | Rural | 1 |
|  |  | Urban | 2 |
| 108 | What is your father educational status? | Can’t read and write | 1 |
|  |  | Can read and write | 2 |
|  |  | Primary | 3 |
|  |  | Secondary | 4 |
|  |  | Diploma and above |  |
| 109 | What is your mother educational status? | Can’t read and write | 1 |
|  |  | Can read and write | 2 |
|  |  | Primary | 3 |
|  |  | Secondary | 4 |
|  |  | Diploma and above | 5 |
| 110 | How many family members in your house? | ------ in number |  |
| 111 | Is there any family member who had mental illness | Yes | 1 |
|  |  | No | 2 |
| 112 | What is your monthly personal income in year? | ……….. |  |
| 113 | Do you have financial distress/problem for stationary (like photocopy) and recreational activity | Yes | 1 |
|  |  | No | 2 |
| 114 | Feeling of insecurity in your safety? | Feel secure | 1 |
|  |  | Feel insecure | 2 |
| 115 | Do you have previous exposure for violence? | Yes | 1 |
|  |  | No | 2 |
| 116 | Do you have financial support | Yes | 1 |
|  |  | No | 2 |
| 117 | Who is your main source of financial support? | Parents | 1 |
|  |  | Siblings | 2 |
|  |  | Self | 3 |
|  |  | Relatives | 4 |
|  |  | Others | 5 |
| 118 | Do you have boy or girl friend? | Yes | 1 |
|  |  | No | 2 |
| 119 | Have you conflict with your boy or girl friend? | Yes | 1 |
|  |  | No | 2 |

**The Self-Reporting Questionnaire (SRQ)**

| Please tick the **‘Yes’** box if you have had this symptom in the **last 30 days** and tick the **‘No’** box if you had not**.** | | | |
| --- | --- | --- | --- |
| 201 | Do you often have headache? | No | Yes |
| 202 | Is your appetite poor? | No | Yes |
| 203 | Do you sleep badly? | No | Yes |
| 204 | Are you easily frightened? | No | Yes |
| 205 | Do your hands shake? | No | Yes |
| 206 | Do you feel nervous? | No | Yes |
| 207 | Is your digestion poor? | No | Yes |
| 208 | Do you have trouble thinking clearly? | No | Yes |
| 209 | Do you feel unhappy? | No | Yes |
| 210 | Do you cry more than usual? | No | Yes |
| 211 | Do you find it difficult to enjoy your daily activities? | No | Yes |
| 212 | Do you find it difficult to make decisions? | No | Yes |
| 213 | Is your daily work suffering? | No | Yes |
| 214 | Are you unable to play a useful part in life? | No | Yes |
| 215 | Have you lost interest in things? | No | Yes |
| 216 | Do you feel that you are a worthless person? | No | Yes |
| 217 | Has the thought of ending your life been on your mind? | No | Yes |
| 218 | Do you feel tired all the time? | No | Yes |
| 219 | Do you have uncomfortable feelings in the stomach? | No | Yes |
| 220 | Are you easily tired? | No | Yes |

**Questions on Academic related Factor**

| 301 | From which department you are? | Nursing | 1 |
| --- | --- | --- | --- |
|  |  | Public health | 2 |
|  |  | Midwifery | 3 |
|  |  | Pharmacy | 4 |
|  |  | Nutrition | 5 |
|  |  | Med. Laboratory | 6 |
|  |  | Environmental Health | 7 |
|  |  | HIT | 8 |
|  |  | Medicine | 9 |
|  |  | Radiology | 10 |
|  |  | Anesthesia | 11 |
| 302 | What is your year of study | 1^st^ year | 1 |
|  |  | 2^nd^ year | 2 |
|  |  | 3^rd^ year | 3 |
|  |  | 4^th^ year | 4 |
|  |  | 5^th^ year | 5 |
| 303 | Department choice | Preferred | 1 |
|  |  | Not Preferred | 2 |
| 304 | Do you have Interest to the department you are learning | Yes | 1 |
|  |  | NO | 2 |
| 305 | Do you ever had serious conflict with instructors | Yes | 1 |
|  |  | No | 2 |
| 306 | Do you have enough vacation time (semester break) | Yes | 1 |
|  |  | No | 2 |
| 307 | Did your grade decrease than expected | Yes | 1 |
|  |  | No | 2 |

**Social support questionnaire**

|  | **QUESTIONS** | **Very Strongly disagree** | **Strongly disagree** | **Disagree** | **Neutral** | **Agree** | **Strongly agree** | **Very Strongly agree** |
| --- | --- | --- | --- | --- | --- | --- | --- | --- |
| **401** | There is a special person who is around when I am in need. | (1) | (2) | (3) | (4) | (5) | (6) | (7) |
| **402** | There is a special person with whom I can share my joys and sorrows. | (1) | (2) | (3) | (4) | (5) | (6) | (7) |
| **403** | My family really tries to help me. | (1) | (2) | (3) | (4) | (5) | (6) | (7) |
| **404** | I get the emotional help and support I need from my family. | (1) | (2) | (3) | (4) | (5) | (6) | (7) |
| **405** | I have a special person who is a real source of comfort to me. | (1) | (2) | (3) | (4) | (5) | (6) | (7) |
| **406** | My friends really try to help me. | (1) | (2) | (3) | (4) | (5) | (6) | (7) |
| **407** | I can count on my friend when things go wrong. | (1) | (2) | (3) | (4) | (5) | (6) | (7) |
| **408** | I can talk about my problems with my family. | (1) | (2) | (3) | (4) | (5) | (6) | (7) |
| **409** | I have friends with whom I can share my joys and sorrows. | (1) | (2) | (3) | (4) | (5) | (6) | (7) |
| **410** | There is a special person in my life who cares about my feelings. | (1) | (2) | (3) | (4) | (5) | (6) | (7) |
| **411** | My family is willing to help me in make decisions. | (1) | (2) | (3) | (4) | (5) | (6) | (7) |
| **412** | I can talk about my problems with my friends. | (1) | (2) | (3) | (4) | (5) | (6) | (7) |

**SUBSTANCE USE (ABUSE)**

| Please tick the **‘Yes’** box if you have had use and **‘No’** box if you don’t use the mentioned substance in your life time or in the last 1 month as directed. | | | |
| --- | --- | --- | --- |
| 501 | Have you used Khat in the last 1 month? | No | Yes |
| 502 | Have you used any kind of alcohol drinks in the last 1 month? | No | Yes |
| 503 | Have you used any kind of tobacco product in the last 1 month? | No | Yes |
| 504 | Have you used any other substances / Such as hashish, and Heroin in the last 1 month? | No | Yes |
