# Supplementary material for: The magnitude of mental distress and associated factors among a school of medicine and college of health sciences students at Debre Markos University, 2021
Source: PLoS One. 2022 Sep 28;17(9):e0275120. doi: 10.1371/journal.pone.0275120 (PMC9518916; doi:10.1371/journal.pone.0275120)
Supplement: Attachment — Submitted filename: Authors point by point Response.docx [file pone.0275120.s003.docx]

**Author’s Point-by-Point Response to the Reviewer's and Editors Reports**

**The magnitude of mental distress and associated factors among A school of medicine and college of health sciences students at Debre Markos university, 2021**

Corresponding Authors Dejen Tsegaye/ [dejenetsegaye@gmail.com](mailto:dejenetsegaye@gmail.com)

**Point by point response to Reviewers and Editors**

First and foremost, the authors would like to express their gratitude to the PLOSE ONE Journal editors and reviewers for thoroughly evaluating this work and offering the required corrections. We made changes based on the feedback we received and presented each comment point by point. The authors attempted to address all of the concerns expressed by the editorial board and reviewers. Please note that the response was written in blue font.

**Authors' responses to the editors' remarks**

**Response to the financial issue:** This work was not funded in any way by the author(s). We also included it in the main document and in the cover letter.

**Response to the Acknowledgement section:** Wolkite University was mentioned as a source of financial assistance. However, this did not imply that Wolkite University had supported our research; it was mentioned in error. The necessary changes to the main document have been made, and our cover letter has been attached.

**Response to the data availability issue:** The manuscript and its supporting information files contain all necessary data. We apologize for the inconvenience; we corrected the error based on your suggestion. It meant that the data used to complete this task was uploaded, and that other data that was not uploaded, such as SPSS data, might be uploaded if asked.

Response to figure 2 reference: Figure 2 has now been appropriately cited. I apologize for the oversight.

**Authors' responses to the authors' remarks**

**REVIEWER #1**

**ABSTRACT:**

**Comment:** Introduction is too long; it should be short. The instrument used to assess mental distress in this study should be mentioned.

**Response:** Thank you for your advice. We made the necessary changes.

**Comment: Key words:** There were only three keywords, which may be increased.

**Response:** Thank you for your suggestions. We have added one additional keyword that is crucial.

**Comment: Introduction:** At the end of the introduction, state the purpose of the study.

**Response:** It is critical to include the study's goal. Thank you for your insightful suggestion. We've added a paragraph that explains it.

METHODS:

Comment: Line-144: "Study participants were not excluded from this study." This is not an exclusion criteria.

**Response:** We didn't have any criteria in place to reject students from the research because of this. Critically ill students, students on summer break, and those with a history of mental illness were all eliminated from the study as a result of your suggestion.

Comment: Line-146: Earlier, no objectives were mentioned.

**Response:** We did not include objectives in the manuscript because we followed the journal's guidelines. We've put the objectives in brackets now, as per your advice. Thank you for taking the time to leave such an insightful comment.

Comment: Line 162-163: There is no need to mention the figure title if there is no figure at this point.

**Response:** Thank you for your input; it was inadvertently mentioned there. We've made a change as a result of your idea.

Comment: Line 181: In the definition, what type of "specified substance" should be mentioned?

**Response:** We indicated substances that a student may have taken in the previous month under designated substances. Thank you for providing us with such a useful comment.

Comment: Line 222: The reference/Id number of the ethical approval should be given.

**Response:** Under ethics approval and consent to participants, the ethical approval reference number has been mentioned, thank you. It was HSC/685/16/19.

**RESULTS:**

Comment: Line 238: There is no need to include 'North West Ethiopia' in the Tables title.

**Response:** Thank you for your advice. 'North West Ethiopia' has been omitted from all table tittles.

Comment: Table 1 shows no consistency in the variables and categories. They need to be rearranged (e.g against the age group in the variable column, the religion of the students was mentioned in the category column.

**Response:** Sure, the table needs to be rearranged, and the appropriate adjustments have been made.

Comment:Table-1 is too long

**Response:** Thank you for your kind feedback. The table was indeed excessively long, and variables that were presented in the description session have been deleted from the table, as per your recommendation.

Comment: Line 243-244: ‘Nearly three fourth of the students ……. have interest to their department’ This sentence is perplexing because of the words ‘with their choice ' and ‘have interest '.

**Response:** The remark implied that students in various departments were studying their departments despite their lack of enthusiasm. In any case, we made grammatical corrections.

Comment: Line 257-258: There is no need to mention figure title without a figure.

**Response:** Thank you for your input; it was inadvertently mentioned there. We've made a change as a result of your idea.

Comment: All the figures are mentioned as Figure No-1.

**Response:** On each figure's reference, the appropriate modification is made.

**CONCLUSION:**

Comment: The last paragraph (372–375), not supported by the findings of this study, should be removed from conclusion

**Response:** Based on your insightful comment, the paragraph concerning recommendations (the last paragraph of conclusion) has been delated.

**REFERENCES:**

Comment: Many of the references are not properly written, and page numbers are missing.

**Response:** Thank you for taking the time to leave such an insightful comment. Following our attempt to look over the references, we made the necessary revisions.

**REVIEWER #2**

**Comment 1.** What are the exclusion criteria of the study? It has to be written clearly

**Response:** We updated exclusion criteria based on your and another reviewer's comments, which include students on summer break and those with a history of mental illness. These criteria were not included in the exclusion criteria section since they were already factored into the non-participant rate.

**Comment 2**. There was merging of variables in table 1 see the rows at Educational status of the mother

**Response:** After going over the original paper, we made the necessary changes. When the manuscript was being produced, the correspondent author made a mistake, sorry.

**Comment 3**. Spacing problems for instance table 1 and the next paragraph and below table 2

**Response:** We made equivalent space across the document after all of the comments were fixed.

**Comment 4**. Inappropriate or unimportant part in table 3 column 2 part, please check and correct

**Response**: Yes, the variables in Table 3, column 2 and row 2 were inaccurate, and we fixed them. Thank you very much.

**Comment 5**. The letters and numbers in figures1 has to be given a key

**Response**: We appreciate your thorough inquiry; we corrected them based on your and other reviewers' comments.

**Comment** 6. The fonts in the figures must be similar with the main text

**Response:** It is a valuable comment, and we created it based on it.

**REVIEWER #3**

Authors have work to improve the quality of this study. The discussion part require some improvement. it follows the same fashion just by comparing the findings and then saying lower and higher than this and that. more over some the conclusions and recommendations are not based on the result example; proper time management and the one recommended for the University clinic workers. Overall the current form of manuscript can be suitable for this reputed journal i.e., Plos One with some improvement.

**Response:** We appreciate your suggestion. We did a number of things to improve the quality of the study after reevaluating the original document, starting with English language correction. We also attempted to update the discussion section in order to improve the discussion's flow. Inappropriate/unimportant thoughts are omitted from the conclusion and recommendation sections, since we had a similar request from another reviewer. Thank you so much for your insightful comment, which will assist us in improving the overall document quality.

**REVIEWER #4**

The manuscript reported the magnitude of mental distress and associated factors among a school of medicine and college of health sciences students at a university, which is an important topic
The manuscript was clear, generally well-written, structures and organized.

The Introduction provided a comprehensive background. However, grammatical and spelling mistakes presents. I advise to review the whole manuscripts for this particular point.
The methods section was specific. Details that supports the reproducibility of the research is presented very-well. The following was a concern:

Comment: Why only undergraduate students were included? To me, post graduate might exhibit distress as well and might be sometime more than undergraduates as they could have families, work or other commitments. Any explanation?

**Response:** We agree with you; actually, post-graduate students may be distressed. However, university for postgraduate students is a second exposure, and they may not have the same mental suffering as undergraduate students in terms of the surroundings, finances, interest in their area, feelings of insecurity, and other factors.

Comment: I don’t see there is a need for figure 1

**Response:** We removed it, thank you.

The results section is thorough, well-subdivided and appropriately supported by data analyses.

Thank you for your helpful feedback.

Comment: Please remove Muslim and protestant from age category and mention them as standalone variable- religion.

**Response:** It was a blunder made during the preparation of the manuscript. We received similar feedback from other reviewers as well, and implemented the necessary changes after reevaluating the original document.

Comment: I cannot read under “Educational status of the mother” in page 12; I believe there is missing variables which correspond to the mentioned categories, please review.

**Response:** Certain factors were merged with the educational status variable in table 1. Now that the changes have been made, it is well written.

Comment: Please re-order figures, all are 1? As a reader couldn’t correlate them easily.

**Response:** Thank you very much; that was a complete blunder that has now been rectified.

Comment: NO need for level of social support; can be written in text only.

**Response:** We received a helpful suggestion from a reviewer to shorten the table. Many variables from the tables are now written in text form, as you suggested. Thank you so much for everything.

Comment: The last figure can be easily demonstrated in a pie chart

**Response:** As per your recommendation, we accomplished it in Pichart.

Comment: Proper formatting is needed.

**Response:** After a thorough examination, suitable formatting is applied to the entire manuscript.

Comment: The Discussion section is well-sourced and comprehensive. However, future implications and research are missed.

**Response:** Thank you for your encouraging words. Future implications have been added, which were actually discussed in the recommendation section.

Comment: The Conclusion provide a clear summation and identify the next steps for future research. However, it is lengthy little bit. Try to be more concise

**Response:** Thank you for your kind words. We tried to condense the concluding section by avoiding irrelevant information.

**We appreciate all of the reviewers' and editors' helpful feedback, suggestions, and questions.**

**Thank you,**

**With kind regards!**
