# Supplementary material for: The magnitude of mental distress and associated factors among a school of medicine and college of health sciences students at Debre Markos University, 2021
Source: PLoS One. 2022 Sep 28;17(9):e0275120. doi: 10.1371/journal.pone.0275120 (PMC9518916; doi:10.1371/journal.pone.0275120)
Supplement: Attachment — Submitted filename: Authors point by point Response 2.docx [file pone.0275120.s004.docx]

**Author’s Point-by-Point Response to the Reviewer's and Editors Reports**

**The magnitude of mental distress and associated factors among A school of medicine and college of health sciences students at Debre Markos university, 2021**

Corresponding Authors Dejen Tsegaye/ [dejenetsegaye@gmail.com](mailto:dejenetsegaye@gmail.com)

**Point by point response to Reviewers and Editors**

First and foremost, the authors would like to express their gratitude to the PLOSE ONE Journal editors and reviewers for thoroughly evaluating this work and offering the required corrections. We made changes based on the feedback we received and presented each comment point by point. The authors attempted to address all of the concerns expressed by the editorial board and reviewers. Please note that the response was written in blue font.

**Authors' responses to the editors' remarks**

Editor: As you will see in the reviews, you have done a nice job with the revision. Reviewer #3 has raised some specific and important revisions to be made.

**Response: Thank you! Reviewer three provided us with some very significant feedback, and we made an effort to make the necessary corrections.**

**Authors' responses to the authors' remarks**

**REVIEWER #3**

Comment: 1. General:

There are too many editorial problems along the manuscript which include sentence structure, grammatical and spacing errors. There is also inconsistent use of words and phrases.

Response: The authors made an effort to review the paper and make corrections for grammatical, spacing, and other concerns, such as terms or phrases that were used inconsistently in relation to your feedback.

Comment: 2. Abstract

• On the introduction section of the abstract, it didn't show why the study conducted

Response: I appreciate your advice. We have inserted a clause that clarifies the aim of the investigation.

• On page 1 line 36, replace 'conducted' by 'employed'

Response: Thank you for your advice. We made the necessary changes.

• On the Methods part of the abstract include the study period and the total sample size.

Thank you for your advice. We have added the study period and total sample size.

Comment: 3. Introduction

• The introduction section of this manuscript lacks coherence. You started by defining the problem then show the effect of mental health distress. The factors came later after the effect. I suggest this flow; 1st define the problem◊then come across with associated factors◊finally, state the effects of mental health distress.

Response: Thank you for your advice. We made the necessary changes.

• Page 5, line 100; don’t use the short for ‘SMCHS’ at its first mention.

Response: Thank you for your advice. We made the necessary changes.

Comment: 4. Methods

• Page 5, line 107; enter the work “design” between the words ‘study’ and ‘was’.

Response: Thank you for your advice. We made the necessary changes.

• Page 5, line 109; avoid repetition on the study design.

Response: Thank you for your input; we've made a change as a result of your idea.

• Your study area description lacks REFERENCE(S).

Response: Thank you for your suggestion. We have added a reference.

• On the exclusion criteria, is ‘March’ among the summer months in Ethiopian context? Because you stated, ‘students on summer break are excluded’

Response: When viewed in the context of Ethiopia, March is not summer. However, starting March, departments from the school and college are permitted to leave the university for break early if they were able to complete all of their courses. This university's curriculum is year-based rather than semester-based. I appreciate you spending the time to make such a thoughtful response.

• Page 6, lines 131 & 135; replace ‘Gondar university’ by ‘University of Gondar, Ethiopia’

Response: Thank you for your input; we've made a change as a result of your idea.

• On sample size determination, put the formula.

Response: We have added the formula based on your suggestion. Thank you.

• Did you consider design effect in your sample size determination? In this regard, figure 1 is not clear and needs elaboration. Example: how many students are there for each department per each year? There are also letters like; P, M, N, E, ….., which needs clarification.

Response: The study was unable to take design effect into account. The design effect can be utilized to correct the estimated sampling variance, as is well knowledge. The study took into account/involved all departments/sections, hence a design effect is not required. The stratification and proportional distribution of sample size to each department were simply depicted in the picture. The departments were revealed by the personalities that were referenced there. Its legend is supplied below the figure. I appreciate you spending the time to make such a thoughtful response.

Comment: 5. Results

• Page 7, line 158; remove the last sentence.

Response: Thank you for your advice. We made the necessary changes.

• Page 12, lines 231, 235 and 239; figures captions are misplaced. Please revise it for all.

Response: Thank you. We revised and made the necessary change.

Comment: 6. Discussion

• The discussion part still needs revision. The finding is well compared with other’s work. However, the way you used to justify discrepancies of your findings with previous works is poor. Justification based on the assessment tool used, sample size difference may not be sound and convincing.

Response: Thank you for your advice. We tried to make necessary modifications.

Comment: 7. Conclusion and recommendation

• What you have recommended for different stakeholders based on the significant associated factors you had.

Response: Based on the finding, recommendations are given to the concerned body. Thank you!
Comment: 8. Figure Legend

Figure 2 & 3 are missed and Figure 6 & 7 added which are not cited in the text.

Response: For this feedback, we've made an effort to carefully go over the entire document. I appreciate you spending the time to make such a thoughtful response.

Comment: 9. References

• The reference part is not written appropriately. Most of the references lack journal name, volume, number and page number (Ref. 1, 3, 5, 9, 10, 15, 20, 21, 26, 32, 33,….). There are also references without year publication. Sometimes CAPITAL letter is used (ref.10). Not only these, consider the other issues too like; use of punctuations.

Response: For citation, we used the endnote program; manual referencing was not used. Because of this, references are immediately cited from the software without any modification, which accounts for all of the improper referencing methods discussed above. Now, in response to your comment, we have made the necessary changes. I appreciate your thoughts.

Comment: 10. Figures

• Figure 2 & 5 are not visible well, replace with a better visibility.

Response: Thank you. To improve visibility, we created another figure for each. The visibility has improved recently.

**We appreciate all of the reviewers' and editors' helpful feedback, suggestions, and questions.**

**Thank you,**

**With kind regards!**
